# A Multi-Column CNN Model for Emotion Recognition from EEG Signals

**DOI:** 10.3390/s19214736

**Published:** 2019-10-31

**Authors:** Heekyung Yang, Jongdae Han, Kyungha Min

**Affiliations:** 1 Industry-Academy Cooperation Foundation, Sangmyung University, Seoul 03016, Korea; yhk775206@smu.ac.kr; 2 Department of Computer Science, Sangmyung University, Seoul 03016, Korea

**Keywords:** emotion, EEG, DEAP, CNN, multi-column

## Abstract

We present a multi-column CNN-based model for emotion recognition from EEG signals. Recently, a deep neural network is widely employed for extracting features and recognizing emotions from various biosignals including EEG signals. A decision from a single CNN-based emotion recognizing module shows improved accuracy than the conventional handcrafted feature-based modules. To further improve the accuracy of the CNN-based modules, we devise a multi-column structured model, whose decision is produced by a weighted sum of the decisions from individual recognizing modules. We apply the model to EEG signals from DEAP dataset for comparison and demonstrate the improved accuracy of our model.

## 1. Introduction

Emotion is a kind of conscious or unconscious feeling for a phenomenon or a work. Emotion is expressed through various biological and physical reactions including voice, text, gestures, facial expressions, and biosignals. People cannot conceal their emotions even though they don’t want to reveal them. A reaction to emotion is a very important key for a communication between people.

Emotional reaction plays a very important role to many human-computer interaction (HCI) applications. Recently, many venders present various systems that react to their users without any explicit requests or commands. The systems catch the implicit request from their users by recognizing their emotion. For example, a recently launched TV set automatically controls its contrast and brightness according to the content playing on the TV. When they play a foot game as their content, it increases contrast and brightness, since the user’s emotion is in exciting mode.

To properly recognize emotion from users, video signal catching the facial expressions, audio signals catching the voice and various biosignals including electroencephalography (EEG), electrocardiogram (ECG), electromyogram (EMG), and photoplethysmogram (PPG), respiration pattern (RSP), and galvanic skin response (GSR) are employed. Among them, the biosignals gather high interest for emotion recognition, since they are spontaneous signals, which people cannot control these reactions at their own will. Some people who can keep calm their faces and voices at the change of their emotions cannot conceal their biosignals.

Emotion recognition using these physiological signals is a very interesting research field. Among them, EEG is very frequently-used signals for emotion recognition. Even though EEG presents a relatively precise measure and an easy interface, it suffers from the non-stationary property of the signal. Therefore, the extraction of temporal correlations of spontaneous EEG signals is a key issue for the emotion recognition from EEG signals.

Many researchers presented various machine learning schemes such as support vector machine (SVM) and k nearest neighborhood (kNN) for emotion recognition from biosignals. Recently, deep learning-based schemes replace the traditional machine learning schemes. CNN and RNN-based models are proposed for emotion recognition from EEG signals. However, they still have problems in resolving the non-stationary property of the EEG signals.

**Figure 1:**
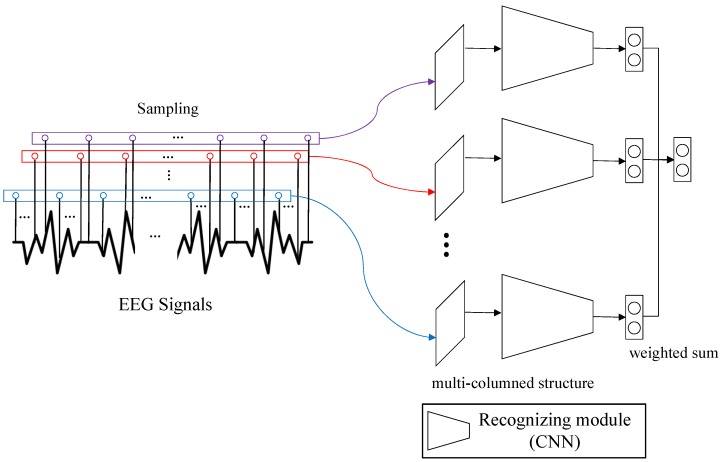
The overview of the algorithm. The EEG signals are sampled at *k* times and each sampled signal is an input to an individual recognizing module. The decisions of the modules are collected to make a final decision.

We present a multi-column CNN-based emotion recognition model from EEG signals (see Figure 1). Our model is composed of several recognition modules that decide the valence and arousal from input EEG signals. The final decision by our model is made by collecting the decisions of individual recognition modules through a weighted voting strategy. Since the individual decision is affected by the non-stationary property of EEG signals, our strategy that makes final decision through a weighted voting can resolve the non-stationary property in a great scale. The experiment on the DEAP dataset demonstrates the excellence of our model.

## 2. Related Work

We categorize the emotion recognition works according to the sources.

### 2.1. EEG-Based Works

#### 2.1.1. Handcrafted Features-Based Works

Brunner et al. [1] compared three independent component analysis (ICA) algorithms including Infomax, FastICA and SOBI to find out to what extent spatial filtering of EEG can improve single trial classification accuracy. After several experiments, they concluded that Infomax outperforms other two ICA variants.

Petrantonakis and Hadjileontiadis [2] employed EEG-based feature extraction scheme for emotion recognition. Their technique is designed using higher order crossings (HOC) analysis which is a robust feature extraction and classification method, which is tested with four classical methods including QDA, kNN, Mahalanobis distance and SVM. As a result, they show that the HOC scheme outperforms their competitors.

Korats et al. [3] conducted a similar experiment by comparing four major ICA algorithms including FastICA, AMICA, Extended Infomax and JADER. In this experiment, AMICA shows an impressive performance.

Duan et al. [4] presented an effective EEG-based emotion classifier based on a feature named differential entropy. They further proposed linear dynamical system (LDS) for feature smoothing method and minimal redundancy maximal relevance algorithm for feature selection algorithm to increase the accuracies and efficiencies of EEG-based emotion classifiers.

Jenke et al. [5] compared 33 studies that extract features for emotion recognition from EEG signals. Their result reveals that features selected by multivariate schemes slightly outperform those from univariate methods.

Zheng [6] proposed a multi channel EEG-based emotion recognition scheme using a novel group sparse canonical correlation analysis (GSCCA), which is a group sparse extension of the conventional CCG method. The GSCCA shows good performance in handing the group feature selection problem from raw EEG features. And, they prove that their GSCCA method outperform the state-of-the-art EEG-based emotion recognition methods.

Mert and Akan [7] employed empirical mode decomposition (EMD) and its multivariate extension (MEMD) for emotion recognition from EEG signals. To resolve the non-stationary behavior and the multichannel issue of EEG signals, they applied MEMD-based feature extraction method to process non-stationary multichannel EEG. They analyzed intrinsic mode functions extracted by MEMD using various time and frequency domain.

#### 2.1.2. CNN-Based Works

Jirayucharoensak et al. [8] presented a deep learning approach for recognizing emotion from nonstationary EEG signals. They employed a stacked autoencoder (SAE) for feature learning. To resolve overfitting problem caused by the non-stationary EEG, they applied PCA to extract the most important component and covariate shift adaptation to minimize the nonstationary effect.

Khosrowabadi et al. [9] presented a six-layer biologically inspired feedforward neural network to discriminate human emotion from EEG signals. The network has a spectral filtering for the input layer, which is followed by a shift register memory. The emotion is discriminated from EEG signals based on valence and arousal levels.

Alhagry et al. [10] developed an LSTM RNN-based emotion recognition technique from EEG signals. The emotions they aim to recognize are in three axes: arousal, valence and liking. They demonstrated accuracy of greater than 85% for the three axes.

Tripathi et al. [11] presented a CNN-based emotion recognition method from EEG signals in the DEAP dataset. They explored two different neural models: a simple deep neural network and a convolutional neural network. The latter demonstrated an improvement of 4.96% than the state-of-the-art techniques.

Salama et al. [12] presented a 3D CNN approach for recognizing emotions from multichannel EEG signals. They developed a data augmentation phase to improve the performance of their 3D CNN model. They employed DEAP dataset to achieve 87.44% accuracy for valence and 88.49% for arousal.

Yang et al. [13] proposed a hybrid neural network combining CNN and RNN to classify human emotion by learning spatial-temporal representation of raw EEG signals. The CNN module mines the inter-channel correation among physically adjacent EEG signals and RNN module mines contextual information of the signals. They tested their model on DEAP dataset and demonstrated that their model achieves 90.80% for valence and 91.03% for arousal.

Moon et al. [14] applied CNN for EEG-based emotion recognition. They employed brain connectivity features, which has not been used in previous studies, to account for synchronous activation of different brain regions. Therefore, their method effectively captures asymmetric brain activity patterns, which plays an important role in emotion recognition.

Li et al. [15] proposed an emotion recognition approach that considers temporal, spatial and frequency characteristics of EEG signals. Their approach extracts RASM as the feature to describe the frequency-space domain characteristic of EEG signals and constructs an LSTM network to explore the temporal correlations of EEG signals. They applied DEAP dataset to their model and achieved 76.67% mean accuracy.

Xing et al. [16] presented a framework that consists of a linear EEG mixing model, which is designed using Stack AutoEncoder, and an emotion timing model, which employes LSTM RNN. Their framework decomposes EEG source signals from collected EEG signals and improves classification accuracy by using the context correlations of the EEG feature sequences. Their model achieved 81.10% accuracy for valence and 74.38% for arousal from DEAP dataset.

### 2.2. Other Sources

#### Facial Expression

Scovanner et al. [17] introduced 3D SIFT descriptor for video, which represents the 3D nature of video data for action recognition. Their scheme discovers relationships between spatio-temporal words in order to describe video data better.

Klaser et al. [18] presented a novel local descriptor based on histograms of oriented 3D spatio-temporal gradients to extract emotion from facial features embedded in video sequences. They applied their descriptor to various action datasets and showed to outperform the state-of-the-art methods.

Liu et al. [19] resolved two important issues including temporal alignment and semantics-aware dynamic representation using manifold modeling of video based on a novel mid-level representation, which is denoted as expressionlet. They modeled expression video clip as a spatio-temporal manifold (STM) and learned a universal manifold model (UMM) for all low-level features. Finally, the local modes on each STM is instantiated by fitting the UMM. Through this approach, the aligned expression video spatially and temporally. They tested their model on four public expression datasets including CK+, MMI, Oulu-CASIA, and AFEW to demonstate that their model outperforms the state-of-the-art methods.

Soleymani et al. [20] presented an emotion recognizing technique based on LSTM RNN and continuous CRF. They annotated the valence from the facial expressions of participants while watching video and measured EEG signals. Both facial expression and EEG are processed on LSTM RNN to extract emotion information.

Zhang et al. [21] combined both EEG and video face signals to recognize human emotions. They proposed a spatial-temporal recurrent neural network (STRNN) that unifies the learning of two different signal sources into a spatial-temporal dependency model.

## 3. Model Construction

**Figure 2:**
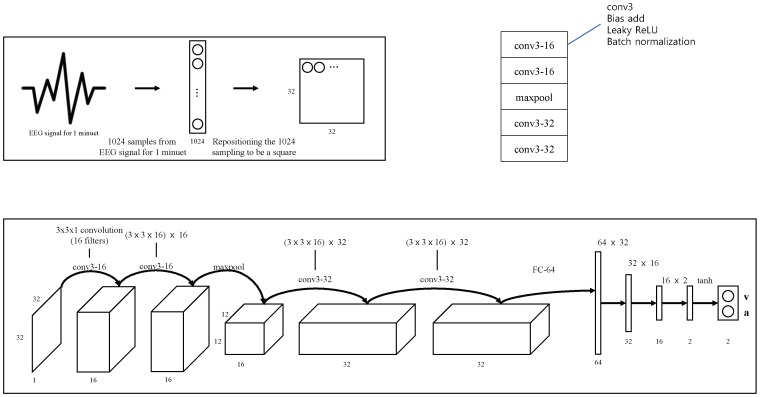
A recognizing module for EEG signal. We sample 1024 discrete values from an EEG signal of DEAP dataset. The sampled values are reformulated to 32×32 rectangular form, which is fed into our CNN-based recognizing module.

We build a multi-column structured model for emotion recognition from EEG signals. Ciresan et al. presented a pioneering multi-column deep neural network model for image classification [22]. This model classifies simple image dataset such as MNIST through a series of identical modules and determines categories by averaging the decisions of the individual modules. Our model is composed of several individually processing recognizing modules. Each recognizing module is designed based on a convolutional neural network (CNN) structure, composed of convolution layers and pooling layers. The structure of a recognizing module is illustrated in Figure 2. The basic building block of the module is a *c*onv3-16, which is composed of conv3 layer followed by bias-add layer. Each module utilizes data from temporal snapshots of DEAP data. The cost function is defined to reflect both class of the emotion and intensity of the emotion. Details of the cost function is:

$$cost\left(arousal,valence\right)\;=\;\left(1-sim_{cos}\right)\left\{\left(a_{i}^{2}-a^{2}\right)+\left(v_{i}^{2}-v^{2}\right)\right\},$$

where $sim_{cos}$ is cosine similarity between projected arousal-valence and actual arousal-valence, both vectorized. The activation function is leacky ReLU. The input, which is reformulated in a rectangular shape, is processed by two *c*onv3-16 layers, followed by a maxpooling layer. After the maxpooling layer, two *c*onv3-32 layers are added. After these layers, four fully connected layers are placed. The final result of these layers are two-fold, each of which corresponds to valence and arousal.

**Figure 3:**
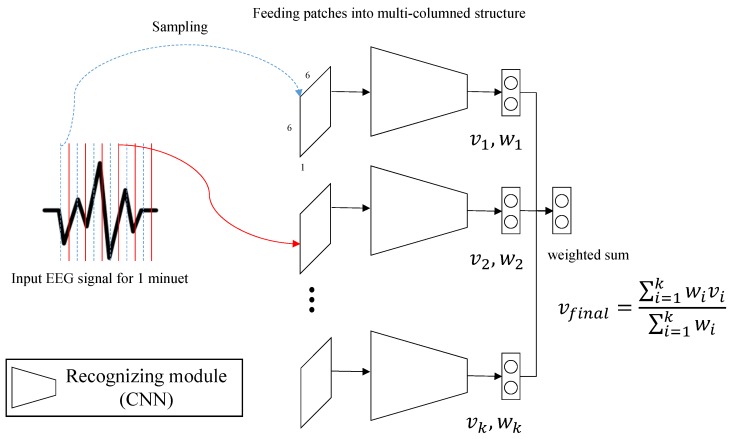
The multi-column structure of our model. The final decision is produced from a weighted sum of the decisions from the individual modules.

The structure of our multi-column model is illustrated in Figure 3. Our model is composed of *k* recognitizing modules illustrated in Figure 2. We vary *k* from 3 to 7 to verify the most effective value. The individual decisions ($v_{i}$) of the modules are merged to a final decision ($v_{final}$) via either voting or a weight sum strategy according to the following formula:

$$v_{final}\;=\;\frac{\sum_{i=1}^{k}w_{i}v_{i}}{\sum_{i=1}^{k}w_{i}},$$

where $v_{final}$ is the final decision of our model, $v_{i}$ is the decision from *i*-th module and $w_{i}$ is the predicted probability for $v_{i}$. $w_{i}$, the weight term for *i*-th decision, comes from the predicted probability of the module. $v_{i}$ is a binary value having either +1 or −1, whereas +1 for high emotion state and −1 for low emotion state. To extract $v_{i}$, we quantize $w_{i}$ in (0.0∼1.0) into nine-point metric in (1∼9). Then, the resulting value following the metric is converted into +1 if it is equal or greater than 5, and −1 if it is less than 5.

The voting strategy follows majority-win rule, which concludes the final decision according to the $v_{i}$ value of majority. Therefore, the final decision $v_{final}$ of the voting strategy is made by the following formula:

$$v_{final}\;=\;\left(\sum_{i=1}^{k}v_{i}>0\right)?+1:-1.$$

In Section 4, we test the accuracies of both strategies and select a better one.

The number of parameters in our model is estimated in Table 1.

## 4. Implementation and Results

### 4.1. Implementation Detail

We have implemented our model in a personal computer with Pentium i7 CPU, 16 GB main memory, and nVidia GeForce TitanX GPU. The model is implemented using Python with Pytorch library.

### 4.2. Data Collection

We employ EEG signal data from DEAP dataset collecting different kinds of physiological signals for human affective state analysis [23]. DEAP dataset contains EEG signals from 32 subjects who watched 40 one-minute music videos. During watching the video, the signals from the subjects are recorded. After watching the video, the subjects rate their emotion in terms of valence, arousal, dominance and liking in nine point metric (1 ∼ 9). We used these ratings as the ground truth for the model. Because we classify emotions with a circumplex model composed with valence and arousal, we excludeed dominance and liking for the research.

**Figure 4:**
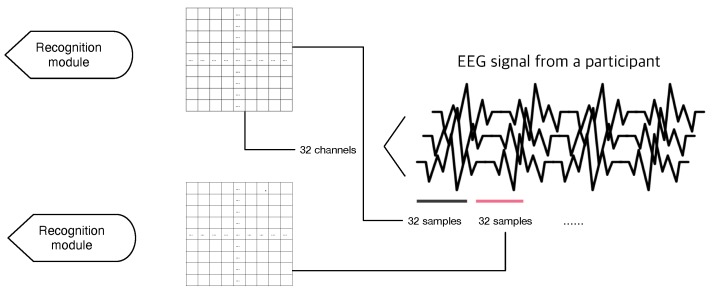
The construction of input data. 32 consecutive samples from 32 EEG channels composite single input vector for a recognizing module. 3–7 modules consume these input data for one training step.

The EEG signals from DEAP dataset are downsampled to 128 Hz and applied by a 4.0–4.5 Hz band-pass filter. Therefore, each trial has 128 × 60 samples for 40 channels, but we exclude 8 channels with supplementary video data due to normalization issue. 32 channels with EEG signal forms one row for our two-dimensional input. 32 consecutive samples form single input data for the model. Figure 4 shows construction of a single input data.

The data from single trial is segmented into (3 + T*k) trial where k denotes the number of columns in our model. The first 3 trials are removed for stability and remaining T trials are for each column.

### 4.3. Model Training

We collect the training data from DEAP dataset in the following strategy. For 32 participants, we collect training dataset from EEG signals of 22 participants, validation dataset from 5 participants, and test dataset from 5 participants. Each participants participated into 40 experiments. We composite a training datum from a participant by sampling 32 consecutive values from equally spaced 7680/(32∗k) different positions of an EEG signal, where k is varying number of the columns consisting our model. Therefore, We collect 33∼80 training data from one EEG signal of a participant for a video, leading into 29,040∼70,400 training dataset for our model. Similarly, we collect each 6600∼16,000 validation dataset and test dataset.

The learning rate for our model is 0.0001, which is reduced by 10 times according to the decrease of the error on validation set. We assign 0.5 for weight decay and 100 for batch size. Our training takes approximately 1 h and 30 min.

### 4.4. Result

We compare the accuracies of our model by comparing the strategy for final decision: voting and weighted sum. We segment the high-state and low-state of valence and arousal by assigning a threshold of 0.5. A valence or arousal whose value is greater than 0.5 is regarded as a high valence or arousal and A valence or arousal less than 0.5 is regarded as a low valence or arousal. We apply a majority-win strategy for voting.

The accuracies for valence and arousal from our model are listed in Table 2, and precision, recall, and F1 score are listed in Table 3. We measure the values by varying the number of columns from 1 to 7.

### 4.5. Comparison

We compare our model with some existing models that recognize emotion from EEG signals. The configuration of our model is 5-column with weighted sum strategy.

**Figure 5:**
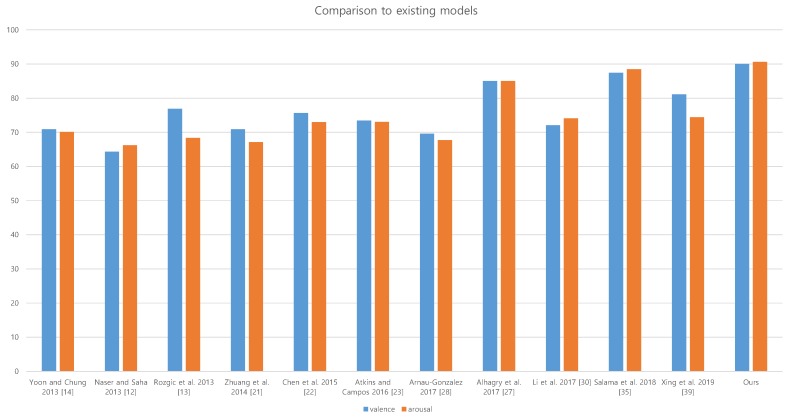
The comparison of our results with the results of existing models.

We compare 11 studies that present emotion recognition techniques from EEG signals in Table 4. Since they measured their performance using DEAP dataset [23], the comparison of the performances is very meaningful. Among them, 7 studies employed handcrafted features and classical recognition framework such as Bayesian network, SVM and HMM. The recent 4 studies employ deep neural network architecture such as CNN and LSTM RNN for recognition framework. We illustrate the result in Figure 5.

The summary of the comparison is suggested in Table 5. In average, the handcrafted feature based works record 71.67% for valence and 69.37% for arousal, while the deep neural network-based works record 81.4% for valence and 80.50% for arousal. The deep neural network-based works record more than 10% higher accuracies. For arousal, the lowest accuracy of deep neural network-based approach, which is 75.12% is higher than the highest accuracy of handcrafted feature-approach, which is 73.06%.

### 4.6. Multi-Class Emotion Recognition

**Figure 6:**
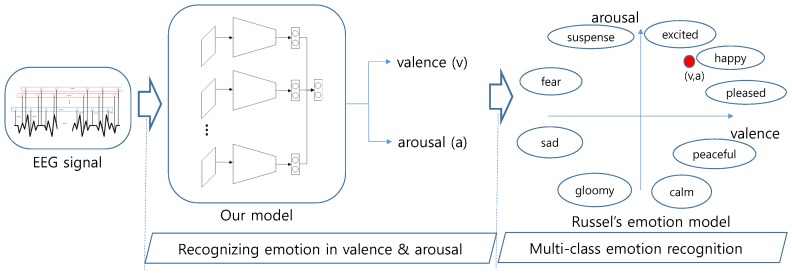
The process of multi-class emotion recognition. The result of our model, which is (valence, arousal), is mapped into the valence-arousal coordinate space of Russell’s model.

Since the result of our model is valence and arousal, the coordinate of valence and arousal in widely-used Russell’s model corresponds to various emotions including *excited*, *happy*, *pleased*, *peaceful*, *calm*, *gloomy*, *sad*, *fear* and *suspense*. We illustrated this process in Figure 6.

### 4.7. Limitation

The most important limitation of our model is a heavy computational load that comes from our multi-column structure. The computational cost for our model increased by the number of columns. Since we employ 5-column models for comparison, the computation time becomes longer than the existing models.

## 5. Conclusions and Future Work

In this paper, we have presented a multi-column structured model for emotion recognition. Each component of our model is an emotion recognizing module based on CNN with various conv, pooling, and fully connected layers. As an input for our model, EEG signal data from DEAP dataset are employed. Our model records higher accuracy than the existing models.

We are going to extend our model to process various biosignals including PPG and GRP. Other features such as facial expressions may be considered. Another direction is to apply our model to compare the emotional responses to real images against those to artworks. 

## Figures and Tables

**Table 1:** The accuracies for valence and arousal with time complexity.

Type of Layer		Filter			Out-Layer	No. of
		Width	Height	In-Layer		Parameters
	conv3-16	3	3	1	16	144
conv	conv3-16	3	3	16	16	2304
	conv3-32	3	3	16	32	4608
	conv3-32	3	3	32	32	9216
	fc-64	12	12	32	64	294,912
fc	fc-32			64	32	2048
	fc-16	-		32	16	512
	fc-2			16	2	32
**Total**						313,776

**Table 2:** The accuracies for valence and arousal with time complexity. **sum** denotes weighted sum.

	Accuracy (%)				Time/	
No. of Columns	Valence		Arousal		Batch Size	Memory (mb)
	Vote	Sum	Vote	Sum	(sec)	
1	84.32		85.18		1.82	2123
3	85.05	85.83	85.90	86.11	2.31	2191
5	89.72	90.01	90.39	90.65	2.88	2240
7	88.45	89.92	90.89	91.00	3.24	2257

**Table 3:** The precision, recall and F1 score.

No.	Precision (%)				Recall (%)				F1 Score (%)			
of	Valence		Arousal		Valence		Arousal		Valence		Arousal	
col.s	vote	sum	vote	sum	vote	sum	vote	sum	vote	sum	vote	sum
1	79.61		80.65		73.68		74.87		76.53		77.65	
3	80.17	81.04	81.27	81.20	75.18	75.72	75.45	76.38	77.59	78.29	78.25	78.72
5	85.20	85.57	86.18	86.66	80.18	80.18	82.07	81.02	82.61	82.79	84.07	83.75
7	84.35	85.64	86.91	86.98	79.54	81.10	82.39	82.87	81.86	83.31	84.59	84.88

**Table 4:** The comparison to existing models. All of these models employ DEAP dataset.

Category	Existing Models	Classifier	Accuracy (%)	
			Valence	Arousal
	Yoon and Chung 2013 [24]	Bayesian	70.90	70.10
	Naser and Saha 2013 [25]	DT-CWPT	64.30	66.20
handcrafted	Rozgic et al. 2013 [26]	decision fusion	76.90	68.40
	Zhuang et al. 2014 [27]	SVM	70.90	67.1
features	Chen et al. 2015 [28]	HMM	75.63	73.00
	Atkins and Campos 2016 [29]	BCI	73.41	73.06
	Arnau-Gonzalez 2017 [30]	SVM	69.60	67.70
	Alhagry et al. 2017 [10]	LSTM RNN	85.00	85.00
	Li et al. 2017 [31]	CRNN	72.06	74.12
Deep learning	Salama et al. 2018 [12]	3D CNN	87.44	88.49
	Xing et al. 2019 [16]	LSTM	81.10	74.38
	Ours	multi-column	90.01	90.65

**Table 5:** Summary of the comparison.

Category		Valence	Arousal
	highest	76.9%	73.06%
handcrafted	lowest	64.3%	66.20%
features	difference	12.6%	6.86%
	average	71.66%	69.37%
	highest	87.44%	88.49%
deep	lowest	72.06%	75.12%
neural network	difference	15.38%	13.37%
	average	81.4%	80.5%
